# Microbiological Quality and Presence of Clinically Relevant Nontuberculous Mycobacteria in Purified Water from Vending Machines in Michoacan, Mexico

**DOI:** 10.3390/pathogens14090886

**Published:** 2025-09-04

**Authors:** Ricardo Jiovanni Soria-Herrera, Janet Karina Hernández-Ramón, Marco Esteban Álvarez-Pérez, Miriam Alejandra Pérez-Sandoval, Margarita Hernandez-Mixteco, Olga Lidia Valenzuela, Eliud Alfredo Garcia-Montalvo, Paola Castillo-Juárez, Sandra Rivera-Gutiérrez, Gilberto Cornejo-Estudillo, Moises León-Juárez, Addy Cecilia Helguera-Repetto, Victoria Campos-Peña, Ma. Guadalupe Zanella-Vargas, Graciela Castro-Escarpulli, Carlos Cortes-Penagos, Jorge Francisco Cerna-Cortés

**Affiliations:** 1 Facultad de Químico Farmacobiología, Universidad Michoacana de San Nicolás de Hidalgo, Morelia 58240, Mexico; ricardo.soria@umich.mx (R.J.S.-H.); qfb.karinahr@gmail.com (J.K.H.-R.); marcoestebanalvarezperez@gmail.com (M.E.Á.-P.); miriam.alejandra.16.08.01@gmail.com (M.A.P.-S.); carlos.cortes@umich.mx (C.C.-P.); 2 Departamento de Microbiología, Escuela Nacional de Ciencias Biológicas, Instituto Politécnico Nacional, Ciudad de México 11340, Mexico; magohdez.m94@gmail.com (M.H.-M.); pcastillo_1307@hotmail.com (P.C.-J.); srivera@ipn.mx (S.R.-G.); chelacastro@hotmail.com (G.C.-E.); 3 Facultad de Ciencias Químicas, Universidad Veracruzana, Orizaba 94340, Mexico; ovalenzuela@uv.mx (O.L.V.); elagarcia@uv.mx (E.A.G.-M.); 4 Escuela José Ma. Pastrana, Secretaría de Educación Pública, Yecapixtla 62829, Mexico; gcornejo2381@gmail.com; 5 Departamento de Inmunobioquimica, Instituto Nacional de Perinatología Isidro Espinosa de los Reyes, Ciudad de México 11000, Mexico; moisesleoninper@gmail.com (M.L.-J.); addy.helguera@inper.gob.mx (A.C.H.-R.); 6 Laboratorio Experimental de Enfermedades Neurodegenerativas, Instituto Nacional de Neurología y Neurocirugía, Manuel Velasco Suárez, Ciudad de México 14269, Mexico; neurovcp@ymail.com; 7 Jurisdicción Sanitaria VI, Secretaria de Salud, Irapuato 36547, Mexico; magzanellav@guanajuato.gob.mx

**Keywords:** purified water, water vending machines, microbiological quality, nontuberculous mycobacteria

## Abstract

In this study, 104 purified water samples were collected from vending machines in the three main cities of Michoacan, Mexico, to assess microbiological quality and the occurrence of diarrheagenic *Escherichia coli* pathotypes (DEP) and nontuberculous mycobacteria (NTM). Aerobic mesophilic bacteria were detected in all samples, with concentrations ranging from 0.95 to 3.71 log_10_ CFU/mL. A total of 62, 34, and 25 samples tested positive for total coliforms, fecal coliforms, and *E. coli*, respectively. Sixty-two samples exceeded Mexico’s official guideline. None of the 58 *E. coli* strains isolated from the 25 *E. coli*-positive samples belonged to DEP. NTM species were recovered from 47 samples, including *M. mucogenicum* (n = 18), *M. abscessus* (n = 11), *M. chelonae* (n = 7), *M. porcinum* (n = 3), *M. fortuitum* (n = 2), *M. septicum* (n = 1), *M. phocaicum* (n = 1), and *M. brisbanense* (n = 1). Three additional isolates could not be identified. All NTM strains produced biofilm and exhibited sliding motility. These findings highlight significant microbiological risks associated with vending machine water and underscore the need for manufacturers to ensure regular maintenance to provide safe and reliable purified water to consumers.

## 1. Introduction

The water vending machine (WVM) market has grown significantly in recent years. The global WVM market was valued at USD 2.65 billion and is projected to reach USD 4.01 billion by 2030. Between 2024 and 2030, the market is expected to grow at a compound annual growth rate of 6.1% [1]. In Mexico, newspapers from several cities have reported an increase of 20–30% in the number of WVMs [2,3]. Many factors influence consumers to obtain purified water from sources other than the tap. However, real and perceived health risks are often the main reason people rely on WVMs for drinking water [4].

**Figure 1:**
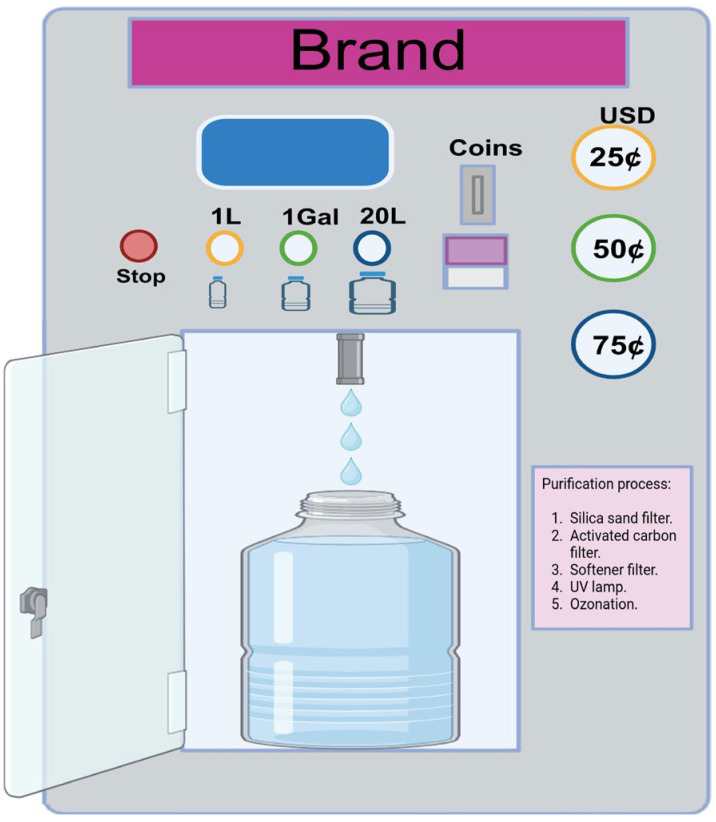
Typical water vending machine.

In Mexico, a WVM (Figure 1) typically refers to a self-service device that dispenses purified water into a container upon insertion of money. These machines are usually located in neighborhoods or public spaces and are widely used as an affordable and accessible source of purified water available 24 h a day, especially in areas where tap water may not be safe to drink. WVMs are designed to improve drinking water quality by reducing organic, inorganic, and bacterial contaminants [5]. However, several studies from different countries have shown that water from vending machines is not necessarily pathogen-free and may contain coliforms and pathogenic bacteria [4,6,7,8]. Therefore, monitoring the water quality of vending machines is essential to ensure that it poses no health risks to consumers.

The term “nontuberculous mycobacteria” (NTM) refers to mycobacteria other than the *Mycobacterium tuberculosis* complex and *M. leprae* [9]. Based on their growth rates, NTM are divided into two groups: slow-growing and rapid-growing species. Rapid growers, such as *M. chelonae* and the *M. abscessus* complex, form visible colonies in less than seven days, whereas slow-growing species, including *M. kansasii* and the *M. avium* complex, take more than seven days to culture [9]. NTM can cause human infections, collectively known as mycobacteriosis, in individuals with specific risk factors that increase vulnerability to these pathogens [10]. Behavioral risk factors include excessive alcohol consumption, exposure to dusty occupations, and smoking. Genetic risk factors include α-1-antitrypsin deficiency, cystic fibrosis, and deficiencies in immune-signaling protein production. HIV infection leading to AIDS is also a major risk factor for NTM infection [11]. Additionally, rare defects in the interleukin-12/interferon-gamma axis and the presence of anti-interferon-gamma autoantibodies may result in disseminated NTM infection [9,10]. Importantly, pulmonary NTM (PNTM) infection also occurs in a distinct group of patients with no apparent underlying cause. These patients are typically tall, lean white women diagnosed with PNTM in their sixth decade of life. They have higher rates of scoliosis, pectus excavatum, mitral valve prolapse, and *CFTR* gene mutations than do controls. Some may also show reduced ciliary beat frequency. The combination of these clinical features and the observation of familial clustering suggests a genetic predisposition to PNTM disease [12]. 

The incidence and prevalence of mycobacteriosis continue to increase worldwide [9,13,14,15]. Approximately 90% of NTM infections affect the lungs, while the remaining cases involve lymph nodes, skin and soft tissue, and bones. Occasionally, NTM can also cause keratitis, otitis media, central nervous system infections, or disseminated infections [9].

NTM have been isolated from a wide range of environmental niches commonly encountered by humans, particularly drinking water. In these environments, NTM can proliferate by utilizing available nutrients, especially when competing microflora are absent or suppressed by disinfectants [16]. Their cell surface hydrophobicity allows them to resist disinfectants and promotes attachment to surfaces, where they can grow and form biofilms [16]. Ingestion of NTM is an important source of infection for individuals with HIV, in whom *M. avium* dissemination often begins with gastrointestinal colonization [9]. One study using DNA fingerprint analysis identified household water as the source of mycobacterial infection in patients with mycobacteriosis [16]. 

To the best of our knowledge, no published study has evaluated the microbiological quality of water from Mexican vending machines, particularly regarding the presence of NTM. We therefore conducted this study to assess microbiological quality and to detect diarrheagenic *Escherichia coli* pathotypes (DEP) and NTM in purified water purchased from vending machines in the three main cities of Michoacan, Mexico, to determine whether this type of water poses a potential health risk to consumers.

## 2. Materials and Methods

### 2.1. Area of Study and Water Collection

**Figure 2:**
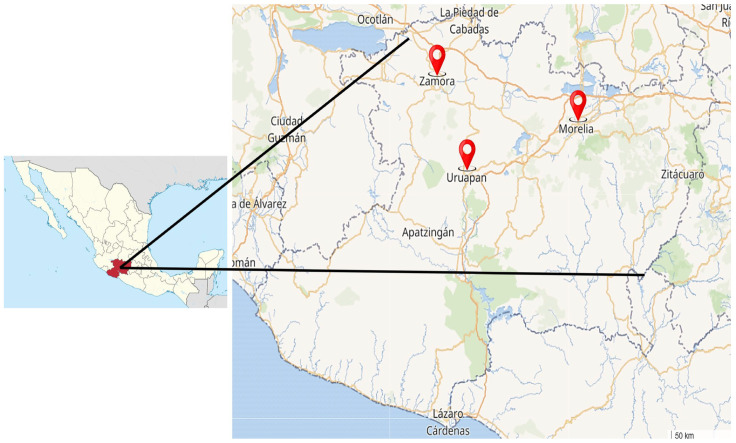
Cities in the state of Michoacan, Mexico, where purified water samples were collected from vending machines: Morelia, Uruapan, and Zamora.

This study was conducted in three main cities in the state of Michoacan, Mexico: Morelia, Uruapan, and Zamora. Together, these cities cover a total area of 1306 km^2^ and have a combined population of approximately 1.5 million inhabitants [17]. Between November 2024 and June 2025, a total of 104 purified water samples were collected from vending machines: 64 from Morelia, 27 from Uruapan, and 13 from Zamora (Figure 2). The WVMs were located in windows or walls of open premises at street level, in areas with high pedestrian traffic, and were accessible to consumers 24 h a day. To ensure that the samples were representative of the water consumed, the outer surface of the vending machines´ tap was not sanitized. The samples were kept below 5 °C during transportation to the laboratory and were analyzed within 6 h from the time of collection. Purified water is defined as potable water that has been subjected to physical or chemical treatment, is free from toxic substances and pathogenic agents, and does not cause adverse health effects when consumed [18]. In WVMs, the purification process typically involves several steps: a silica sand filter (to retain solid waste particles), an activated carbon filter (to remove organic contaminants such as chlorine and volatile organic compounds), a softening filter (to reduce hardness by removing minerals like calcium and magnesium), polishing filters (to remove fine inorganic particles suspended in the water), and finally, a UV lamp and ozonizer (to kill microorganisms, including bacteria and viruses.

### 2.2. Chemical and Microbiological Analysis

One-gallon glass jugs were sterilized prior to sample collection. A pH meter (model pH 209; HANNA Instruments, Sarmeola di Rubano-PD, Italy) and the N,N-diethyl-p-phenylene-diamine technique were used to measure pH and residual chlorine concentrations, respectively [18]. Each sample was analyzed for the presence of aerobic mesophilic bacteria (AMB), total coliforms (TC), fecal coliforms (FC), and *E. coli* following procedures authorized by the U.S. Food and Drug Administration’s *Bacteriological Analytical Manual* [19]. The methods, media, incubation times, and temperatures used for the isolation, identification, and confirmation of ABM, TC, FC, and *E. coli* are provided in Table S1. *Escherichia coli* strains were further examined for the presence of six DEP loci by two multiplex polymerase chain reactions (PCRs) [20,21]. 

All data obtained for this study were analyzed in accordance with the Mexican guideline NOM-201-SSA1-2015 [18]. According to this guideline, purified water must have a pH between 6.6 and 8.5, a free residual chlorine concentration of no more than 0.1 ppm, and no detectable TC (<1.1 most probable number (MPN)/100 mL) in any 100 mL sample.

### 2.3. Isolation and Identification of Mycobacteria

Water samples were processed as previously described by Cerna-Cortes et al. [22] with some modifications. Briefly, to isolate NTM, 500 milliliters of water were decontaminated with 0.5 mL of a 1% cetylpyridinium chloride solution and incubated at room temperature for 30 min. The samples were then filtered using the CORNING® sterile filtration system, which has a membrane of 0.22 μm. Subsequently, the membrane was placed onto Middlebrook 7H10 agar plates (Difco, Becton Dickinson, Franklin Lakes, NJ, USA) supplemented with albumin dextrose catalase (Becton Dickinson BBL™), cycloheximide (500 μg/mL), and the PANTA cocktail (Becton Dickinson BBL™) (40 U/mL polymyxin B, 4 μg/mL amphotericin B, 16 μg/mL nalidixic acid, 4 μg/mL trimethoprim, and 4 μg/mL azlocillin). Plates were incubated at 35 °C. Once the bacterial growth had been observed on the Middlebrook 7H10 agar, the number of colony-forming units ([CFU]/500 mL) was directly determined in each plate, and the identification of acid-fast bacilli was carried out by Ziehl–Neelsen stain. Two established PCR assays were used to identify isolates belonging to the genus *Mycobacterium* and the *M. tuberculosis* complex [23]. Three techniques were applied to identify NTM species: sequencing of a 723 bp fragment of the *rpo*B gene [24], sequencing of the hypervariable region 2 (V2) of the 16S rRNA gene [25], and PCR restriction enzyme pattern analysis of the 65 kDa heat shock protein gene (*hsp*65), as described by Telenti et al. [26]. 

### 2.4. Mycobacterial Biofilm and Sliding Motility Assays

Mycobacterial biofilm formation and motility assays were performed in triplicate. The crystal violet culture plate method was used to assess biofilm production [27], and the level of biofilm formation was scored as absent, weak, moderate, or strong [28]. Mycobacterial spreading was evaluated using the semisolid agar method, and motility was classified as nonmotile or as having low, moderate, or high motility [29]. *Mycobacterium smegmatis* mc^2^ 155 was used as a positive control in both assays. 

### 2.5. Statistical Analyses

Data were summarized using descriptive statistics and are presented in Table 1 as frequencies and percentages. A chi-square (χ^2^) test was applied to compare the frequency of NTM-positive samples across cities. Data normality was evaluated using the Shapiro–Wilk test. The association between the nominal variable (presence of NTM) and the quantitative variables (concentrations of AMB, TC, FC, and *E. coli*) was assessed using the Spearman correlation coefficient (rho). A *p*-value of <0.05 was considered statistically significant. All statistical analyses were performed with Stata 17.0 (StataCorp LLC, College Station, TX, USA).

## 3. Results

### 3.1. Chemical and Microbiological Quality of Purified Water

The pH of the water samples ranged from 6.9 to 7.9, and the chlorine concentration was below 0.1 ppm. Regarding microbiological quality, all 104 samples analyzed were positive for AMB (Table 1). AMB concentrations ranged from 0.95 to 3.71 log_10_ CFU/mL. A total of 62 (59.6%), 34 (32.7%), and 25 (24.0%) water samples tested positive for TC, FC, and *E. coli*, respectively. The TC, FC, and *E. coli* levels ranged from <1.1 to >23.0 MPN/100 mL, <1.1 to 16.1 MPN/100 mL, and <1.1 to 12.0 MPN/100 mL, respectively. Sixty-two water samples containing TC exceeded Mexico’s official guideline (Table 1). Fifty-eight *E. coli* strains were isolated from the twenty-five *E. coli*-positive samples, and none were identified as DEP.

### 3.2. Mycobacteria Isolation and Identification

Of the 104 water samples analyzed, 47 (45.2%) contained NTM, with one strain isolated per sample. Mycobacterial concentrations ranged from 1 to 25 CFU/500 mL. NTM were detected in 51.5%, 40.7%, and 23.0% of water samples from Morelia, Uruapan, and Zamora, respectively (Table 2). All NTM isolates belonged to the rapidly growing mycobacteria group. *Mycobacterium mucogenicum* was the most frequently identified species (18 strains), followed by *M. abscessus* (11 strains), *M. chelonae* (7 strains), *M. porcinum* (3 strains), *M. fortuitum* (2 strains), and one strain each of *M. septicum*, *M. phocaicum*, and *M. brisbanense*. In addition, three isolates could not be identified at the species level (Table 2). The frequency of NTM isolation across cities did not differ significantly (χ^2^ = 12.51, p = 0.819). No correlations were found between the presence of NTM and AMB (rho = −0.0306, p = 0.7579), TC (rho = −0.0133, p = 0.8930), FC (rho = −0.0734, p = 0.4590), or *E. coli* (rho = −0.0597, p = 0.5470).

### 3.3. Mycobacteria Biofilm and Sliding Motility

All NTM strains demonstrated both biofilm formation and sliding motility. Of the 47 strains, 15 were weak biofilm producers, 20 were moderate producers, and 12 were strong producers. Likewise, all strains exhibited sliding motility: 14 showed moderate motility, while the remaining strains displayed high motility (Table 3).

## 4. Discussion

In Michoacan, Mexico, the number of WVMs has been increasing; however, limited microbiological information is available on the water they produce. This study, therefore, evaluated the microbiological quality of purified water from vending machines and assessed the occurrence of NTM species in these samples. 

We analyzed 104 purified water samples collected from vending machines in three cities in Michoacan. The samples had pH values ranging from 6.9 to 7.9 and chlorine concentrations below 0.1 ppm. Thus, all samples were within the chemical standards recommended by the Mexican Official Guidelines for purified water.

Our results show that AMB were present in all water samples, with concentrations ranging from 0.95 to 3.75 log_10_ CFU/mL. These findings are consistent with those reported in Arizona, United States, by Chaidez et al. [4]. By contrast, they differ from the results of Hile et al. [30] in California, who found AMB in only 25% of water samples, with concentrations ranging from 3.83 to 6.5 log_10_ CFU/mL. The AMB count reflects the level of microorganisms in a product and indicates sanitary conditions during storage and handling, as well as the effectiveness of treatment processes [31,32]. High levels of AMB in water, as observed in several of our samples, may suggest the presence of opportunistic pathogens capable of causing infections in immunocompromised individuals [33].

The detection of TC, FC, and *E. coli* in drinking water is a critical indicator of water quality and potential health risks. These bacteria serve as indicator organisms for assessing water safety, particularly with respect to fecal contamination and the possible presence of pathogenic microorganisms [32,34]. In our study, 62 (59.6%), 34 (32.7%), and 25 (24.0%) water samples tested positive for TC, FC, and *E. coli*, respectively. Overall, the 62 TC-positive samples (59.6%) exceeded Mexico’s official guideline. The presence of TC in vending machine water has also been reported in other countries: 20.0% in the United States [4], 38.8% in Malaysia [6], and 94.1% in the United Arab Emirates [35]. Regarding FC and *E. coli*, Al Moosa et al. [35] reported no detections in United Arab Emirates samples, whereas Yougyod et al. [36] identified *E. coli* in 7 (11.1%) of 63 water samples from vending machines in Thailand. In our study, none of the *E. coli* strains belonged to DEP; however, *E. coli* O157:H7 has been detected in vending machine water elsewhere [6]. Positive coliform samples in purified water may indicate treatment inefficiency [37]. It is important to recognize that improper operation of the UV lamp may lead to system failure, allowing a high bacterial load to persist. Moreover, Chaidez et al. [4] reported biofilm formation on the vending machines’ tap, which could serve as an additional contamination source. Therefore, regular maintenance and thorough cleaning of vending machines are essential to reduce or eliminate bacterial contamination. We recommend further studies to monitor water quality both before and after the purification process, given that a substantial proportion of samples were found to be outside guideline values. Moreover, indicator bacteria should be eliminated from purified water by applying effective disinfectants and robust purification procedures.

Of note, Mexican health authorities are required to conduct sanitary surveillance of purified water producers to monitor compliance with NOM-201-SSA1-2015 [18], NOM-251-SSA1-2009 [38], and NOM-127-SSA1-2021 [39], as stipulated in Article 400 of the General Health Law. In practice, however, this verification often cannot proceed because no owner or employee is present at the establishment to receive the inspection. We therefore recommend that legislators amend the relevant provisions of the General Health Law and the Federal Administrative Procedure Law to require producers to be available for inspections. This would help ensure the proper functioning of vending machines and allow corrective actions to be taken when necessary to protect consumers.

NTM can cause severe mycobacteriosis in humans [40]. In this study, 47 (45.2%) of 104 water samples contained NTM. All NTM identified in this study have been associated with human diseases [41,42,43,44,45]. Skin and soft tissue infections are primarily caused by *M. fortuitum*, *M. abscessus*, and *M. chelonae* [46]. Notably, *M. mucogenicum*, the most prevalent NTM in this study, has been linked to gastrointestinal tract infection in a patient with Crohn’s disease [47]. Future studies employing DNA fingerprinting are needed to determine whether the NTM isolated from vending machine water are genetically identical to those isolated from patients. 

The presence of NTM in water samples can be explained by the fact that these organisms are commonly found in natural and engineered water systems, where they thrive in biofilms and resist conventional disinfection methods [16]. NTM are also highly resistant to chlorine and UV radiation. Depending on the species, the UV dose required to achieve the same level of inactivation as *E. coli* is significantly higher. Schiavano et al. [48] reported that inactivating *Mycobacterium avium* subsp. *hominissuis* required a UV dose 480% greater than the upper limit of the range recommended by international standards for drinking water disinfection (16–40 mJ/cm^2^). Detection of only one mycobacterial species per sample may be attributable to the methodology employed. Future studies should utilize multiplex real-time PCR/melting curve analysis or whole-genome sequencing (WGS) to enable the simultaneous identification of multiple mycobacterial species present in water samples.

Of note, *M. avium* was not detected in the water samples. This result is consistent with Cerna-Cortés et al. [22], who did not detect *M. avium* in purified water samples from Mexico City. Although evidence confirms that NTM are present in drinking water, the numbers and recovery frequencies of *M. avium* and *M. intracellulare* in purified water are generally low [49]. These authors also reported that higher concentrations of *M. avium* in water are associated with increased turbidity and particulate matter, likely due to bacterial attachment to particles. Furthermore, water treatment processes substantially reduce mycobacterial loads in raw water by 2 to 4 log units. These factors may explain the absence of *M. avium* in the samples analyzed.

All NTM isolates produced biofilm and exhibited sliding motility. The ability to form biofilms, which protect against external pressures, is a key factor in the persistence of mycobacteria in the environment. Biofilms enable long-term colonization of new substrates, including human tissues [40], and provide NTM strains with an added barrier against exposures such as antimicrobial agents and disinfectants (e.g., chlorine) [50]. Sliding motility, however, is crucial for NTM surface colonization in both environmental and host settings [29]. Eliminating mycobacteria from purified water is challenging because of their robust cell structure, capacity for biofilm formation, resistance to conventional disinfection methods, and ability to survive in low-nutrient environments [16]. Therefore, effective technological innovations in WVMs are urgently needed to eliminate NTM from purified water.

## 5. Conclusions

Our findings show that most purified water samples contained coliform bacteria, and nearly half harbored NTM associated with human disease. Regular inspections by the appropriate authorities are therefore essential to enforce legislation on microbiological standards for purified water and to reduce the risk of gastrointestinal and other illnesses linked to its consumption. These results are also critical for manufacturers, who must ensure that WVMs are properly maintained in order to provide safe and reliable drinking water to the public.

## Figures and Tables

**Table 1:** Quantity and frequencies of AMB, TC, and FC in purified water samples from vending machines ^a.^

Microorganism Group	Minimum	Median	Maximum	Frequency (%)	Number of Samples Outside of the 201 Guideline * (%)
AMB	0.95	1.64	3.71	104 (100)	NA
TC	<1.1	1.1	>23	62 (59.6)	62 (59.6)
FC	<1.1	<1.1	16.1	34 (32.7)	NA
*E. coli*	<1.1	<1.1	12	25 (24.0)	NA

^a^ n = 104. Minimum, median, and maximum values are presented as log_10_ CFU/mL for AMB and as MPN/100 mL for TC, FC, and *E. coli*. * Guideline requires that TC must not be detectable in any 100 mL sample (<1.1 MPN/100 mL). AMB, aerobic mesophilic bacteria; TC, total coliforms; FC, fecal coliforms; MPN, most probable number; NA, not applicable.

**Table 2:** NTM species identified in water samples from vending machines.

Isolate Code	Species of NTM Identified ^a^ (Total)	Morelia No. of Strains	Uruapan No. of Strains	Zamora No. of Strains
12, 17, 19, 22, 25, 26, 27, 28, 29, 30, 31, 32, 33, 36, 38, 39, 42, and 46	*M. mucogenicum* (n = 18)	13	3	2
6, 7, 9, 13, 18, 24, 34, 37, 40, 41, and 43	*M. abscessus* (n = 11)	7	4	-
2, 15, 16, 21, 44, 45, and 47	*M. chelonae* (n = 7)	4	3	-
1, 5, and 35	*M. porcinum* (n = 3)	3	-	-
4 and 10	*M. fortuitum* (n = 2)	1	1	-
20	*M. septicum* (n = 1)	1	-	-
3	*M. phocaicum* (n = 1)	1	-	-
23	*M. brisbanense* (n = 1)	1	-	-
8, 11, and 14	*M.* sp. (n = 3)	2	-	1
	No. of NTM / No. of samples collected (%)	33/64 (51.5%)	11/27 (40.7%)	3/13 (23.0%)

NTM = Nontuberculous mycobacteria. ^a^ = 47 NTM identified. - = not isolated.

**Table 3:** Biofilm production and sliding motility of NTM strains.

		Biofilm Production ^a^			Sliding Motility ^b^		
NTM Species	No. De Strains	Weak	Moderate	Strong	Low	Moderate	High
*M. mucogenicum*	5			+			+
*M. mucogenicum*	3		+				+
*M. mucogenicum*	3		+			+	
*M. mucogenicum*	3	+					+
*M. mucogenicum*	2			+		+	
*M. mucogenicum*	2	+				+	
*M. abscessus*	5		+				+
*M. abscessus*	2		+			+	
*M. abscessus*	2			+			+
*M. abscessus*	2	+					+
*M. chelonae*	3	+					+
*M. chelonae*	1	+				+	
*M. chelonae*	1		+				+
*M. chelonae*	1			+			+
*M. chelonae*	1			+		+	
*M. porcinum*	1	+					+
*M. porcinum*	1	+				+	
*M. porcinum*	1		+				+
*M. fortuitum*	1		+				+
*M. fortuitum*	1		+			+	
*M. septicum*	1		+				+
*M. phocaicum*	1	+					+
*M. brisbanense*	1	+					+
*M.* sp.	2		+				+
*M.* sp.	1			+		+	

^a^ Optical density limits at 570 nm for biofilm production: nonproducer, ≤0.103; weak producer, 0.103–0.207; moderate producer, 0.207–0.413; strong producer, >0.413. ^b^ Sliding motility zones: nonmotile, ≤5 mm; low motility, 6–9 mm; moderate motility, 10–15 mm; high motility, >15 mm.

## Data Availability

All data derived from this study are provided in this article.

## Supplementary Materials

The following supporting information can be downloaded at: https://www.mdpi.com/article/10.3390/pathogens14090886/s1, Table S1: Listing of methods of detection, identification, confirmation, and reporting of results of microorganisms in purified water from WVMs.

## Abbreviations

The following abbreviations are used in this manuscript:

AMB	Aerobic mesophilic bacteria
DEP	Diarrheagenic *Escherichia coli* pathotypes
FC	Fecal coliforms
MPN	Most probable number
NTM	Nontuberculous mycobacteria
PCR	Polymerase chain reaction
TC	Total coliforms
WVM	Water vending machine
