# Supplementary material for: Microbiological Quality and Presence of Clinically Relevant Nontuberculous Mycobacteria in Purified Water from Vending Machines in Michoacan, Mexico
Source: Pathogens. 2025 Sep 4;14(9):886. doi: 10.3390/pathogens14090886 (PMC12472464; doi:10.3390/pathogens14090886)
Supplement: Supplementary file 1 [file pathogens-14-00886-s001.zip › pathogens-3856469-supplementary.pdf]

pathogens

MDPI

Table S1. Listing of methods of detection, identification, confirmation, and reporting of results of microorganisms in purified water from WVMs

Microorganisms	Presumptive test or primary Enrichment	Confirmatory test or secondary Enrichment	Selective medium for isolation	Identification test	Results are reported as
Aerobic mesophilic bacteria	NR[a]	Plate count agar (Bioxon™, BD, State of Mexico, Mexico) for 24-48h at 37 °C.	NR	NR	CFU/mL[c]
Total coliforms	Lactose broth (Difco™, BD, Sparks, MD) for 48h at 37 °C	Brilliant green lactose bile broth (DifcoTM, BD, Sparks, MD, USA) for 48 h at 35°C	NR	Gas production	MPN/100 mL[b]
Fecal coliforms	Lactose broth (Difco™, BD, Sparks, MD) for 48h at 37 °C	*Escherichia coli* broth (Difco™, BD, Sparks, MD) for 48h at 44.5 °C	NR	Gas production	MPN/100 mL[b]
Escherichia coli	Lactose broth (Difco™, BD, Sparks, MD) for 48h at 37 °C	*Escherichia coli* broth with MUG (4-methylumbelliferyl-β-D-glucuronide) (Difco™, BD, Sparks, MD) for 48h at 44.5 °C	Eosin methylene blue agar (Bioxon™, BD, State of Mexico, Mexico) for 24h at 37 °C	Indole production, Methyl red test, Voges-Proskauer test, and citrate utilization (Bioxon™, BD, State of Mexico, Mexico) for 24h at 37 °C	MPN/100 mL[b]

[a] NR, Not Required.

[b] MPN/100 mL, most probable number per 100 mL.

[c] CFU/mL, colony forming units per mL.
